# Objects in Contact with Classical Scrapie Sheep Act as a Reservoir for Scrapie Transmission

**DOI:** 10.3389/fvets.2015.00032

**Published:** 2015-09-14

**Authors:** Timm Konold, Stephen A. C. Hawkins, Lisa C. Thurston, Ben C. Maddison, Kevin C. Gough, Anthony Duarte, Hugh A. Simmons

**Affiliations:** ^1^Animal Sciences Unit, Animal and Plant Health Agency Weybridge, Addlestone, UK; ^2^Pathology Department, Animal and Plant Health Agency Weybridge, Addlestone, UK; ^3^Surveillance and Laboratory Services, Animal and Plant Health Agency Penrith, Penrith, UK; ^4^ADAS UK, School of Veterinary Medicine and Science, University of Nottingham, Sutton Bonington, UK; ^5^School of Veterinary Medicine and Science, University of Nottingham, Sutton Bonington, UK

**Keywords:** classical scrapie, prion, transmissible spongiform encephalopathy, sheep, field furniture, reservoir, serial protein misfolding cyclic amplification

## Abstract

Classical scrapie is an environmentally transmissible prion disease of sheep and goats. Prions can persist and remain potentially infectious in the environment for many years and thus pose a risk of infecting animals after re-stocking. *In vitro* studies using serial protein misfolding cyclic amplification (sPMCA) have suggested that objects on a scrapie-affected sheep farm could contribute to disease transmission. This *in vivo* study aimed to determine the role of field furniture (water troughs, feeding troughs, fencing, and other objects that sheep may rub against) used by a scrapie-infected sheep flock as a vector for disease transmission to scrapie-free lambs with the prion protein genotype *VRQ/VRQ*, which is associated with high susceptibility to classical scrapie. When the field furniture was placed in clean accommodation, sheep became infected when exposed to either a water trough (four out of five) or to objects used for rubbing (four out of seven). This field furniture had been used by the scrapie-infected flock 8 weeks earlier and had previously been shown to harbor scrapie prions by sPMCA. Sheep also became infected (20 out of 23) through exposure to contaminated field furniture placed within pasture not used by scrapie-infected sheep for 40 months, even though swabs from this furniture tested negative by PMCA. This infection rate decreased (1 out of 12) on the same paddock after replacement with clean field furniture. Twelve grazing sheep exposed to field furniture not in contact with scrapie-infected sheep for 18 months remained scrapie free. The findings of this study highlight the role of field furniture used by scrapie-infected sheep to act as a reservoir for disease re-introduction although infectivity declines considerably if the field furniture has not been in contact with scrapie-infected sheep for several months. PMCA may not be as sensitive as *VRQ/VRQ* sheep to test for environmental contamination.

## Introduction

Classical scrapie is a transmissible spongiform encephalopathy (TSE) of sheep and goats, which is caused by infection with a proteinacious infectious particle, the prion (1). Prions are particularly resistant to commonly used disinfectants and inactivating methods and can remain biologically active, which increases the risk of re-infection after re-stocking.

Soil has been implicated as plausible reservoir of scrapie prions because laboratory studies have shown that scrapie-associated prion protein (PrP^Sc^) can bind to soil particles, which may remain close to the surface where it was released so that it is accessible to grazing animals (2). Indeed, pastures not used by a scrapie-infected flock for more than a month remained contaminated and led to infection of genetically fully susceptible “clean” sheep, even if exposed for only 6 weeks (3). However, the scrapie agent was reportedly also able to re-infect sheep in a building that had not been used for 16 years (4); thus, reservoirs other than soil contribute to scrapie transmission.

While contamination of the environment had historically been mainly attributed to prions in placentae from scrapie-infected ewes (5), it has now been established by PrP^Sc^ detection methods that various sources may contribute to environmental contamination, such as urine (6), feces (7), saliva (8), and possibly rubbed off skin (9), which explains the lateral transmission of scrapie to sheep where no lambing has occurred (10, 11). However, less is known about the vectors that facilitate disease transmission, particularly in buildings that have previously housed scrapie-infected sheep.

Prions were detected by serial protein misfolding cyclic amplification (sPMCA) in swabs taken from water troughs and fences that had been present on a scrapie-infected sheep farm, and it was hypothesized that they could act as environmental reservoirs to contribute to scrapie transmission (12). The current study was designed to investigate the persistence of environmental scrapie infectivity using a sheep bioassay. As there was surprisingly little evidence of environmental contamination on pastures used previously by a scrapie-infected flock after re-introduction of new sheep, this study aimed to confirm the relevance of field furniture as a vector for scrapie transmission by exposing sheep to objects that had been in contact with a scrapie-infected flock.

## Materials and Methods

All procedures were in accordance with the United Kingdom (UK) Animal (Scientific Procedures) Act 1986, under license from the UK Government Home Office (project licenses 70/6774 and 70/7782) following approval by the institutional ethical review process.

### Scrapie-infected flock

A scrapie research flock was established in 1998 by purchasing clinically healthy sheep from scrapie-affected flocks with scrapie-susceptible genotypes and maintained through breeding sheep of susceptible genotypes (10). At the time of the project, more than 50% of the sheep in the flock were homozygous *VRQ* (valine at codon 136, arginine at 154, and glutamine at 171 of the ovine prion protein gene), which is associated with high susceptibility to classical scrapie (13), and developed clinical disease from 18 months of age. Sheep were kept on pastures for many years, being kept in sheds just before lambing and occasionally in severe weather. The pasture was managed on a rotation basis and was plowed and reseeded as required. Manure and composted bedding from the lambing sheds was incinerated and not spread on the pasture. For logistical reasons, sheep with clinical signs suggestive of scrapie were predominantly kept on one particular pasture to enable closer monitoring until cull at clinical end-stage.

### Classical scrapie-free flock

Testing for infectivity was carried out using Cheviot sheep from a flock that was originally generated from imported sheep from New Zealand and kept free of classical scrapie through strict biosecurity measures (14), which was confirmed routinely by testing of culled sheep’s brains for TSEs (Bio-Rad TeSeE; Bio-Rad Laboratories, UK). All sheep were homozygous *VRQ*.

### Level of contamination of field furniture in clean housing

Field furniture that had been in contact with the scrapie flock 8 weeks earlier was used. Selection of items was to some degree influenced by the results from an earlier study where environmental sources of scrapie prions were investigated by sPMCA and which had demonstrated the presence of PrP^Sc^ on a fire extinguisher box (referred to as plastic scratching post in Reference 12), wooden fence post, and fencing (12) 2 years earlier. Sample extractions from objects and sPMCA methodology were carried out as described previously (12). Briefly, eight swabs were taken for each surface, a swab sample consisted of a wetted foam swab that had been rubbed 10 times over an area of approximately 10 cm × 2 cm, and these were stored frozen at −80°C until extraction. Samples were extracted using a silicon dioxide enrichment methodology (15), the extracts from two swabs being finally eluted in 200 μl of 0.1% w/v SDS solution, of which 10 μl was then used to seed each sPMCA reaction. Each extracted sample was amplified in triplicate or duplicate reactions for nine sPMCA rounds using a substrate consisting of a 10% healthy *VRQ/VRQ* sheep brain homogenate [a different brain substrate to that used in Ref. (12)]. The 0.2-ml PCR tubes containing the extracts and substrate were placed in an ultrasonicating water bath (model S4000; Misonix, USA) at 37°C. Sonications were performed for 40 s at 200 W and these were repeated once every 30 min for 24 h (one PMCA round). After each PMCA round, the samples were diluted 1 in 3 with fresh substrate in a final volume of 100 μl and the process repeated up to a total of nine rounds. Reaction products were then digested with 50 μg/ml proteinase K (PK) with the addition of 0.045% (wt/vol) SDS for 1 h at 37°C before western blot analysis using 12% (wt/vol) NuPAGE precast Bis-Tris gels. Reactions were scored positive if a PK-resistant triplet was visible on the western blot after probing with the antibody SHa31- and HRP-based chemiluminescent detection.

For *in vivo* testing of the infectivity of objects from pastures grazed by the scrapie flock, 24 *VRQ/VRQ* lambs 1–5 days of age (83% aged 2 days) were housed in a building that had never been used for any classical scrapie-infected sheep. The lambs, which were kept with their dams until 3–4 months of age, were housed in four pens with group sizes determined by availability of sheep and housing capacity as follows:

Pen 1. Five lambs in a pen with clean furniture (building controls).
Pen 2. Six lambs in a pen with a water trough from a pasture used mainly by subclinically infected sheep of the scrapie flock.
Pen 3. Six lambs in a pen with a water trough from a pasture, which was used by clinically affected sheep prior to cull.
Pen 4. Seven lambs in a pen with a wooden fence post, fencing with traces of fleece from sheep attached to it and a fire extinguisher box. This box had been fixed outside an area where the sheep from the scrapie-infected flock would gather just before entering the handling area and had been used by sheep to rub their backs and head.

None of the items were cleaned but the water troughs were emptied and filled with new water in the animal accommodation. All items placed into pens 2–4 were swabbed and analyzed by sPMCA in triplicate before arrival of the sheep. Swabs were taken from analogous surfaces to the scrapie-contaminated farm, e.g., water troughs, metal hurdles, etc., at the farm housing the classical scrapie-free flock to serve as control samples, which were tested in parallel with the samples from the scrapie-contaminated objects.

The pens shared common air space but were separated by 3 m high concrete walls, and each pen had its own entrance and separate equipment and protective clothing to avoid cross-contamination.

Scrapie infection was monitored by rectal biopsy at approximately 6, 9, 13, and 19 months post exposure (mpe). Biopsies were taken under local anesthesia with a prilocaine and lidocaine mixture (EMLA cream; AstraZeneca, UK) and the recto-anal mucosa-associated lymphoid tissue (RAMALT) examined for presence of disease-associated prion protein (PrP^Sc^) with rat monoclonal antibody R145 (APHA, UK) according to previously published methods (16). For the rectal biopsy at 6 mpe, lambs were sedated with 0.8 ml acepromazine (ACP injection 2 mg/ml; Novartis Animal Health, UK) given intramuscularly approximately 30 min prior to the biopsy due to their restlessness. Sheep were examined for signs of scrapie according to a short clinical protocol (17) prior to cull from 23 mpe, whereby sheep were assessed by a veterinarian for signs of abnormal behavior (e.g. separation from others), gait (e.g. ataxia), and sensation (e.g. reaction to scratching with head or lip movements, impaired menace response) and – depending on the display of signs – classified as clinical suspect, inconclusive, or clinically healthy with regards to scrapie.

Scrapie infection was determined postmortem by immunohistochemical examination of lymphoreticular tissue (RAMALT, distal ileum, mesenteric lymph node) in all animals and – in all sheep over 12 months of age – additional examination of brain tissue (right and left half of the obex) for PrP^Sc^ by immunohistochemistry (18) and for the proteinase-resistant form of PrP^Sc^ (PrP^res^) by ELISA (Bio-Rad TeSeE; Bio-Rad Laboratories, UK) according to the manufacturer’s instructions.

### Exposure of sheep to contaminated furniture on pasture in paddock 1

For the field study, a 5.4 ha-sized pasture that had been occupied by scrapie sheep was divided into four equal paddocks, with two paddocks (termed 1 and 2) used for the reported study. Each paddock, measured approximately 13,000 m^2^, was double fenced with new fencing, with a minimum of 2 m between the fences to prevent nose-to-nose contact between sheep in different paddocks. Each paddock had its own set of protective clothing for farm workers and equipment.

Contamination of the soil in both paddocks used for the reported study was previously investigated by moving groups of six 2-day-old lambs with their dams to these paddocks, each equipped with a new water trough. None of the six lambs in each group had detectable PrP^Sc^ in a rectal biopsy taken at 6 and 9 mpe and although they were subsequently moved to a different paddock at 10 mpe, none of the sheep presented with detectable PrP^Sc^ in brain and lymphoid tissue when culled at 34 months of age.

Contaminated field furniture added to paddock 1, which by that time had not been grazed by scrapie-affected sheep for 40 months, were metal hurdles, a metal lamb creep and a water trough, replacing the previous one, which all had been in contact with the scrapie flock 8 weeks earlier.

The paddock was occupied by 24 lambs aged 1–4 days (67% aged 2 days) with their dams, with the exception of one ewe that arrived with a 14-day-old lamb delivered by cesarean section. Dams were removed 3–4 months later and not tested for scrapie. Rectal biopsies were taken at approximately 9 mpe and examined as described above. Based on the RAMALT results (see Results), all sheep were culled from 11 mpe and examined for scrapie by postmortem tests as before.

Swabs were taken as described above from water trough, fence, wooden post, hurdles, and lamb creep 5 days after the first sheep were introduced in the paddock and examined for PrP^Sc^ by sPMCA in duplicate reactions.

### Effect of replacement of contaminated furniture on pasture in paddock 1

Twelve 18- to 28-day-old lambs (median 21 days) were moved with their dams to paddock 1, which had been occupied up to 7 days previously by sheep to test for infectivity of contaminated furniture (see above), but clean furniture was now provided (water trough from a paddock not used by scrapie sheep before, fencing replaced). The increase in age was due to poor weather conditions at the time of lambing. Dams were removed as before after 3–4 months. The sheep were culled from 11 mpe and tissues examined by TSE postmortem tests, as described above.

### Exposure of sheep to weathered contaminated furniture on pasture in paddock 2

Concurrently with the furniture replacement study, 13 12- to 25-day-old lambs (median 21 days) moved with their dams to paddock 2, which had been grazed by the natural scrapie flock 53 months previously, and contained a water trough and additional objects (lamb creep and hurdles used during lambing of the scrapie flock) from the scrapie-infected flock, which had not been used by these sheep for 18 months. Removal of dams and TSE testing protocol was identical to the furniture replacement study.

### Statistical tests

Where appropriate infection rates between groups were compared by Fisher’s exact test (Prism 6, GraphPad Software, USA), with *P* < 0.05 classified as statistically significant (adjusted in the case of multiple comparisons by multiplying the *P* value following each test by the number of comparisons).

## Results

The results of the sheep studies are summarized in Table 1.

**Table 1:** **Summary of the results in each sheep study**.

Location	Exposure to	Rate of infection (*N* positive/*N* total)
**Level of contamination of field furniture not used by scrapie flock for 8 weeks**		
Pen 1	Clean furniture (control)	0/5
Pen 2	Water trough (used by predominantly pre-clinical scrapie sheep)	0/6
Pen 3	Water trough (used also by clinically affected scrapie sheep)	4/5^a^
Pen 4	Scratch post, fence, fire extinguisher box	4/7
**Exposure of sheep to contaminated furniture on pasture**		
Paddock 1^b^	Metal hurdles, metal lamb creep, water trough not used by scrapie flock for 8 weeks on pasture not used by scrapie flock for 40 months	20/23^c^
**Effect of replacement of contaminated furniture on pasture**		
Paddock 1	Water trough not used by scrapie sheep before and new fencing on pasture not occupied by pre-clinical scrapie sheep for a week	1/12
**Exposure of sheep to weathered contaminated furniture on pasture**		
Paddock 2^b^	Water trough, lamb creep and hurdles, not used by the scrapie flock for 18 months on pasture not occupied by the scrapie flock for 53 months	0/13

*^a^One lamb was excluded, which died 28 days post exposure and was not TSE tested*.

*^b^No scrapie infection demonstrated in groups of six lambs occupying these paddocks for up to 10 months, after they had been used by the scrapie flock 5 months earlier*.

*^c^One lamb was excluded, which was culled 7 days post exposure due to joint infection and not TSE tested*.

### Level of contamination of field furniture in clean housing

Infection of sheep was first demonstrated in RAMALT at 9 mpe in a water trough-exposed group (water trough previously exposed to clinically infected sheep) and at 13 mpe in the rubbing objects-exposed group. Scrapie was confirmed in four sheep on brain examination by immunohistochemistry (three exposed to the water trough, one to rubbing objects) but only one was also confirmed by ELISA. This sheep was the only sheep that presented with clinical signs prior to cull at 23 mpe. Evidence of infection in the other sheep was based on presence of PrP^Sc^ in lymphoid tissue only.

Despite evidence of infection in the sheep exposed to one of the water troughs and the scratch objects, the difference compared to the control group was statistically not significant (*P* = 0.14 and *P* = 0.24, respectively, after adjusting for multiple comparisons).

PrP^Sc^ was detected by sPMCA in swabs taken from water trough and fencing (see Table 2) in an assay that could consistently detect PrP^Sc^ within 10 pg of ovine brain from a clinically affected animal. However, PrP^Sc^ was also detected in 2 out of 139 reactions from environmental swab samples taken from the classical scrapie-free farm, and the difference in the sPMCA results between scrapie-affected and scrapie-free farm was statistically not significant (*P* = 0.124).

**Table 2:** **Detection of PrP^Sc^ by sPMCA in swabs from objects placed in clean housing and on pasture**.

Location	Object	sPMCA result (*N* positive/*N* total)
**Level of contamination of field furniture not used by scrapie flock for 8 weeks**		
Pen 2	Water trough used mainly by pre-clinical scrapie cases	
	Sample 1	0/3
	Sample 2	0/3
Pen 3	Water trough used by clinically affected scrapie cases	
	Sample 1	0/3
	Sample 2	1/3
Pen 4	Fire extinguisher box	0/3
	Fencing with traces of fleece attached to it	
	Sample 1	0/3
	Sample 2	1/3
	Fence post	
	Sample 1	0/3
	Sample 2	0/3
**Exposure of sheep to contaminated furniture on pasture**		
Paddock 1	Water trough	0/2
	Fence	0/2
	Wooden post	0/2
	Hurdles	0/2
	Lamb creep	0/2

### Exposure of sheep to contaminated furniture on pasture in paddock 1

**Figure 1:**
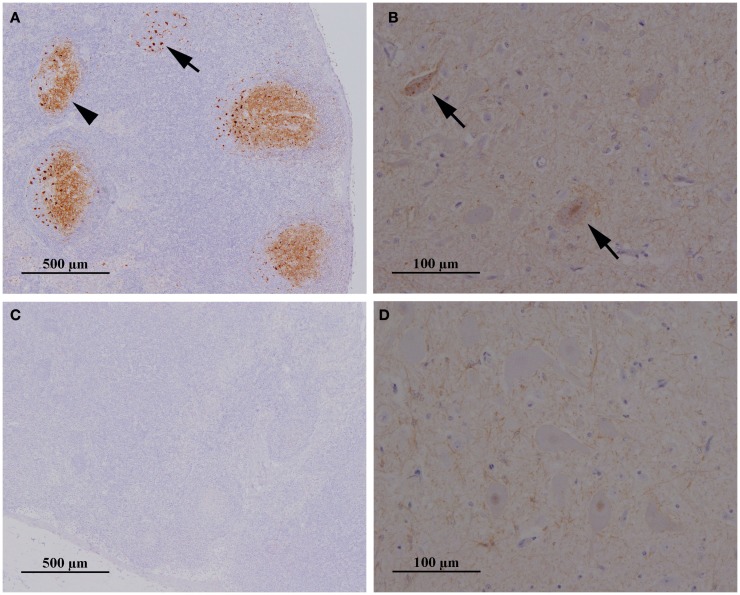
**Presence and absence of PrP^Sc^ in the lymph node and brainstem of two sheep exposed to contaminated furniture on pasture in paddock 1**. Immunohistochemical examination of mesenteric lymph node **(A,C)** and brainstem at the level of the obex **(B,D)** taken from sheep 25/13 **(A,B)** and 38/13 **(C,D)**, respectively. Antibody: R145. Sheep 25/13 presents with PrP^Sc^ in tingible body macrophages **(A)**, black arrow, and the network of follicular dentritic cells **(A)**, black arrowhead, in follicles of the lymph node and also in neurons in the dorsal motor nucleus (parasympathetic nucleus) of the vagus nerve **(B)**, black arrows, whereas PrP^Sc^ is neither detectable in lymph node **(C)** nor in brainstem **(D)** of sheep 38/13.

PrP^Sc^ was first detected in RAMALT of 18 (78%) sheep. When the sheep were culled 3 months later at 11–12 mpe, scrapie was confirmed by postmortem tests on lymphoid tissue in 20 (87%), six of which also had PrP^Sc^ in the obex (no detectable PrP^res^ by ELISA). Figure 1 gives an example of a sheep with confirmed scrapie infection compared to a sheep in the same paddock with no evidence of infection.

None of the swabs taken from the furniture yielded detectable PrP^Sc^ by sPMCA (Table 2), even though other analogous environmental samples (from inside farm buildings) run concurrently for a different study yielded positive results (data not presented).

### Effect of replacement of contaminated furniture on pasture in paddock 1

Scrapie infection was confirmed in a single sheep (8%), which presented with PrP^Sc^ in lymphoid tissue only. Compared with exposure of sheep to contaminated furniture in the same paddock, this was a significant reduction in the infection rate (*P* < 0.0001).

### Exposure of sheep to weathered contaminated furniture on pasture in paddock 2

Exposure to objects not used by scrapie-infected sheep for 18 months did not result in any infection.

## Discussion

Classical scrapie is an environmentally transmissible disease because it has been reported in naïve, supposedly previously unexposed sheep placed in pastures formerly occupied by scrapie-infected sheep (4, 19, 20). Although the vector for disease transmission is not known, soil is likely to be an important reservoir for prions (2) where – based on studies in rodents – prions can adhere to minerals as a biologically active form (21) and remain infectious for more than 2 years (22). Similarly, chronic wasting disease (CWD) has re-occurred in mule deer housed in paddocks used by infected deer 2 years earlier, which was assumed to be through foraging and soil consumption (23).

Our study suggested that the risk of acquiring scrapie infection was greater through exposure to contaminated wooden, plastic, and metal surfaces via water or food troughs, fencing, and hurdles than through grazing. Drinking from a water trough used by the scrapie flock was sufficient to cause infection in sheep in a clean building. Exposure to fences and other objects used for rubbing also led to infection, which supported the hypothesis that skin may be a vector for disease transmission (9). The risk of these objects to cause infection was further demonstrated when 87% of 23 sheep presented with PrP^Sc^ in lymphoid tissue after grazing on one of the paddocks, which contained metal hurdles, a metal lamb creep and a water trough in contact with the scrapie flock up to 8 weeks earlier, whereas no infection had been demonstrated previously in sheep grazing on this paddock, when equipped with new fencing and field furniture. When the contaminated furniture and fencing were removed, the infection rate dropped significantly to 8% of 12 sheep, with soil of the paddock as the most likely source of infection caused by shedding of prions from the scrapie-infected sheep in this paddock up to a week earlier.

This study also indicated that the level of contamination of field furniture sufficient to cause infection was dependent on two factors: stage of incubation period and time of last use by scrapie-infected sheep. Drinking from a water trough that had been used by scrapie sheep in the predominantly pre-clinical phase did not appear to cause infection, whereas infection was shown in sheep drinking from the water trough used by scrapie sheep in the later stage of the disease. It is possible that contamination occurred through shedding of prions in saliva, which may have contaminated the surface of the water trough and subsequently the water when it was refilled. Contamination appeared to be sufficient to cause infection only if the trough was in contact with sheep that included clinical cases. Indeed, there is an increased risk of bodily fluid infectivity with disease progression in scrapie (24) and CWD (25) based on PrP^Sc^ detection by sPMCA. Although ultraviolet light and heat under natural conditions do not inactivate prions (26), furniture in contact with the scrapie flock, which was assumed to be sufficiently contaminated to cause infection, did not act as vector for disease if not used for 18 months, which suggest that the weathering process alone was sufficient to inactivate prions.

PrP^Sc^ detection by sPMCA is increasingly used as a surrogate for infectivity measurements by bioassay in sheep or mice. In this reported study, however, the levels of PrP^Sc^ present in the environment were below the limit of detection of the sPMCA method, yet were still sufficient to cause infection of in-contact animals. In the present study, the outdoor objects were removed from the infected flock 8 weeks prior to sampling and were positive by sPMCA at very low levels (2 out of 37 reactions). As this sPMCA assay also yielded 2 positive reactions out of 139 in samples from the scrapie-free farm, the sPMCA assay could not detect PrP^Sc^ on any of the objects above the background of the assay. False positive reactions with sPMCA at a low frequency associated with *de novo* formation of infectious prions have been reported (27, 28). This is in contrast to our previous study where we demonstrated that outdoor objects that had been in contact with the scrapie-infected flock up to 20 days prior to sampling harbored PrP^Sc^ that was detectable by sPMCA analysis [4 out of 15 reactions (12)] and was significantly more positive by the assay compared to analogous samples from the scrapie-free farm. This discrepancy could be due to the use of a different sPMCA substrate between the studies that may alter the efficiency of amplification of the environmental PrP^Sc^. In addition, the present study had a longer timeframe between the objects being in contact with the infected flock and sampling, which may affect the levels of extractable PrP^Sc^. Alternatively, there may be potentially patchy contamination of this furniture with PrP^Sc^, which may have been missed by swabbing. The failure of sPMCA to detect CWD-associated PrP in saliva from clinically affected deer despite confirmation of infectivity in saliva-inoculated transgenic mice was associated with as yet unidentified inhibitors in saliva (29), and it is possible that the sensitivity of sPMCA is affected by other substances in the tested material. In addition, sampling of amplifiable PrP^Sc^ and subsequent detection by sPMCA may be more difficult from furniture exposed to weather, which is supported by the observation that PrP^Sc^ was detected by sPMCA more frequently in indoor than outdoor furniture (12). A recent experimental study has demonstrated that repeated cycles of drying and wetting of prion-contaminated soil, equivalent to what is expected under natural weathering conditions, could reduce PMCA amplification efficiency and extend the incubation period in hamsters inoculated with soil samples (30). This seems to apply also to this study even though the reduction in infectivity was more dramatic in the sPMCA assays than in the sheep model. Sheep were not kept until clinical end-point, which would have enabled us to compare incubation periods, but the lack of infection in sheep exposed to furniture that had not been in contact with scrapie sheep for a longer time period supports the hypothesis that prion degradation and subsequent loss of infectivity occurs even under natural conditions.

In conclusion, the results in the current study indicate that removal of furniture that had been in contact with scrapie-infected animals should be recommended, particularly since cleaning and decontamination may not effectively remove scrapie infectivity (31), even though infectivity declines considerably if the pasture and the field furniture have not been in contact with scrapie-infected sheep for several months. As sPMCA failed to detect PrP^Sc^ in furniture that was subjected to weathering, even though exposure led to infection in sheep, this method may not always be reliable in predicting the risk of scrapie infection through environmental contamination. These results suggest that the VRQ/VRQ sheep model may be more sensitive than sPMCA for the detection of environmentally associated scrapie, and suggest that extremely low levels of scrapie contamination are able to cause infection in susceptible sheep genotypes.

## Author Contributions

TK analyzed the data and drafted the manuscript. HS designed and managed the study, supported by SH, LT, and TD who participated in the coordination of different parts of the study. BM and KG were responsible for the sPMCA investigations. All authors contributed to the final draft of this manuscript and read and approved the final manuscript.

## Conflict of Interest Statement

The authors declare that the research was conducted in the absence of any commercial or financial relationships that could be construed as a potential conflict of interest.

## Funding

The study was funded by the UK Department for Environment, Food and Rural Affairs (project SE1861).
